# STAT3, the Challenge for Chemotherapeutic and Radiotherapeutic Efficacy

**DOI:** 10.3390/cancers12092459

**Published:** 2020-08-30

**Authors:** Ping-Lian Yang, Lu-Xin Liu, En-Min Li, Li-Yan Xu

**Affiliations:** 1 The Key Laboratory of Molecular Biology for High Cancer Incidence Coastal Chaoshan Area, Shantou University Medical College, Shantou 515041, Guangdong, China; yang_pinglian@grmh-gdl.cn (P.-L.Y.); 18lxliu2@stu.edu.cn (L.-X.L.); 2 Institute of Oncologic Pathology, Shantou University Medical College, Shantou 515041, Guangdong, China; 3 Department of Biochemistry and Molecular Biology, Shantou University Medical College, Shantou 515041, Guangdong, China

**Keywords:** chemoresistance, radioresistance, STAT3, STAT3β, target therapy

## Abstract

**Simple Summary:**

STAT3, an oncogene, contributes to insensitivity of chemotherapy and radiotherapy in tumor, reduces the clinical efficacy. Meanwhile, STAT3β, a STAT3 splicing isoform, is related to the inhibition of tumor growth and chemosensitivity. STAT3 may become a potential target to overcome the chemo(radio)resistance, which benefit for developing novel drugs targeting STAT3 or alternative splicing regulators.

**Abstract:**

Chemoradiotherapy is one of the most effective and extensively used strategies for cancer treatment. Signal transducer and activator of transcription 3 (STAT3) regulates vital biological processes, such as cell proliferation and cell growth. It is constitutively activated in various cancers and limits the application of chemoradiotherapy. Accumulating evidence suggests that STAT3 regulates resistance to chemotherapy and radiotherapy and thereby impairs therapeutic efficacy by mediating its feedback loop and several target genes. The alternative splicing product STAT3β is often identified as a dominant-negative regulator, but it enhances sensitivity to chemotherapy and offers a new and challenging approach to reverse therapeutic resistance. We focus here on exploring the role of STAT3 in resistance to receptor tyrosine kinase (RTK) inhibitors and radiotherapy, outlining the potential of targeting STAT3 to overcome chemo(radio)resistance for improving clinical outcomes, and evaluating the importance of STAT3β as a potential therapeutic approach to overcomes chemo(radio)resistance. In this review, we discuss some new insights into the effect of STAT3 and its subtype STAT3β on chemoradiotherapy sensitivity, and we explore how these insights influence clinical treatment and drug development for cancer.

## 1. Introduction

Chemoradiotherapy is the main treatment for various solid malignancies through the induction of tumor DNA damage, and it can be used alone or in combination with surgery. Because of the genetic and epigenetic heterogeneity of cancer, the response to chemoradiotherapy is variable, and accompanied by therapeutic toxicities and resistances that limit the effectiveness of chemoradiotherapy [1,2,3,4]. In recent years, many reports have demonstrated that STAT3 contributes to resistance to chemotherapy and radiotherapy [5,6,7].

**Figure 1:**
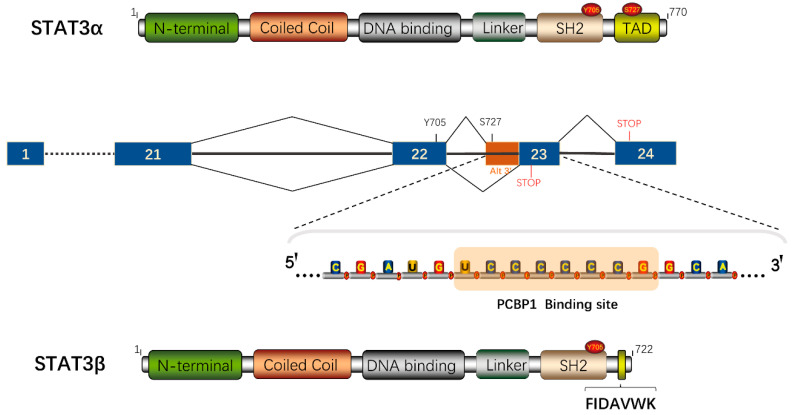
Schematic diagram of the structure and the alternative splicing pattern of STAT3. PCBP1 binds to “UCCCCCCG” and promotes the usage of an alternative 3’ splicing site in exon 23, which increases the expression of STAT3β. The TAD (transcriptional activation domain) of STAT3α is replaced by FIDAVWK.

Signal transducer and activator of transcription (STAT) proteins are a family of cytoplasmic transcription factors and were discovered by James E. Darnell while investigating interferon signal transduction [8]. Seven mammalian members (STAT1, STAT2, STAT3, STAT4, STAT5a, STAT5b and STAT6) contribute to multiple biological functions, including cell differentiation, development, proliferation, apoptosis and inflammation [9]. STAT3 shares a common structure with other STAT proteins, and consists of an amino-terminal domain (NTD) that is required for cooperative DNA binding [10], a coiled-coil domain (CCD) for STAT3 recruitment to a receptor, a DNA-binding domain (DBD), a linker domain, an Src homology 2 (SH2) domain, which is involved in STAT3 dimerization, and a C-terminal transcriptional activation domain (TAD) for transcription activation (Figure 1) [11,12].

STAT3 is identified as an acute-phase response factor that is activated in response to IL-6, growth factors and hormones [13,14,15,16,17]. Once STAT3 Tyr705 is phosphorylated by receptor tyrosine kinases (JAK) or non-receptor tyrosine kinases (Src, Abl), STAT3 homodimerizes or heterodimerizes through its SH2 domain; it then translocates into the nucleus and binds to its recognition GAS (TTCN_2-4_GAA) elements in target genes to modulate gene transcription [18,19]. STAT3β is an alternatively spliced variant, in which the 55 C-terminal amino acid residues of STAT3α, including serine 727, are replaced by seven amino acid residues (FIDAVWK). Poly-C binding protein-1 (PCBP1) can interact with an exonic splicing suppressor in STAT3 exon 23 by binding to the sequence “UCCCCCCG”, and can then regulate the proportion of STAT3α/STAT3β (Figure 1) [20]. STAT3β is generally considered a dominant-negative regulator of STAT3α. However, it still acts as a significant transcriptional regulator for its own specific genes [21]. Constitutively-activated STAT3 has been frequently detected in human cancers and contributes to cancer progression [22,23,24,25,26,27,28,29,30]. STAT3 activation is also associated with maintaining embryonic stem cell self-renewal. While STAT3 deficiency causes early embryonic lethality, STAT3β itself can rescue the embryonic lethality [31,32,33]. 

Herein, this review focuses on the mechanism of STAT3 in resistance to chemo(radio)therapy and improvement in the therapeutic effect of chemoradiotherapy (CRT)-resistant tumors by targeting STAT3.

## 2. Feedback Loop Leading to STAT3 Activation

**Figure 2:**
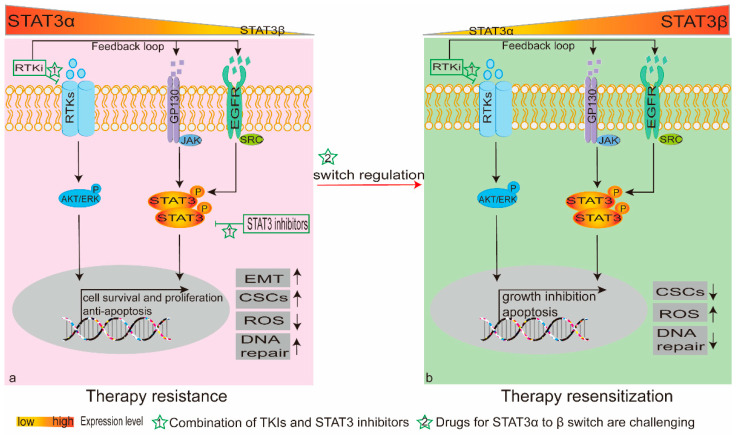
Schematic representation of different ratios of STAT3α/STAT3β and their functions in therapy sensitivity. (**a**) The STAT3 signaling pathway confers therapy resistance to cells, mainly by mediating its target genes for cell survival, proliferation and inhibition of apoptosis. It promotes epithelial-mesenchymal transition, restores cancer stem cell properties, enhances DNA repair and attenuates ROS levels. RTK inhibitors can augment cell survival and proliferation via feedback activation of STAT3. Thus, in combination with RTK inhibitors, STAT3 inhibitors can be an effective strategy for therapy resistance. (**b**) STAT3β activation is better for cell growth inhibition and apoptosis, in contrast with STAT3α functions. Switch regulation for STAT3α to STAT3β is a challenging approach for resensitization to chemotherapy. In the context of a high expression level of STAT3β, RTK inhibitors that activate STAT3 via a feedback loop may be of benefit for cell apoptosis. Abbreviations: AKT, protein kinase B; CSCs, cancer stem cells; EGFR, epidermal growth factor receptor; EMT, epithelial-mesenchymal transition; JAK, Janus kinase; P, phosphorylation; PCBP1, poly-C binding protein-1; ROS, reactive oxygen species; RTKi, receptor tyrosine kinase inhibitor; RTKs, receptor tyrosine kinases; STAT3, signal transducer and activator of transcription 3.

Mammalian cells use various regulatory mechanisms, involving feedback loops and crosstalk between signaling pathways, to adjust to perturbations in cellular homeostasis [34]. These mechanisms are pivotal in determining cell fate under dynamic physiological circumstances and pose a barrier to the effective therapy of cancer. The development of small-molecule inhibitors has significantly improved progression-free survival against receptor tyrosine kinases (RTKs), but disappointing outcomes remain due to the emergence of drug resistance (Figure 2). Interestingly, erlotinib, as an RTK inhibitor, increases STAT3 phosphorylation (Tyr705) in EGFR-mutant ono-small-cell lung cancer (NSCLC) cells through an autocrine loop, whereas knockdown of STAT3 decreases erlotinib-resistant colony numbers in the presence of erlotinib. This suggests that the interruption of a STAT3 feedback loop blocks chemotherapy resistance [35]. A similar result was reported for HER2-overexpressing breast and gastric cancers, where hyperactivated STAT3 signaling-mediated trastuzumab resistance occurred via a feedback loop comprising FN/EGF/IL-6 upstream mediators [36]. Moreover, MEK inhibitor induced STAT3 feedback activation, leading to resistance in KRAS mutant lung cancer cells [37]. MEK blockage has poor clinical outcome in KRAS-mutant (KRASMT) colorectal cancer cells, and siRNA-mediated knockdown of macrophage inhibitory factor (MIF) restored sensitivity to refametinib by decreasing STAT3 phosphorylation [38]. In addition, combined STAT3 and BCR-ABL1 inhibition can attenuate BCR-ABL1 kinase-dependent TKI resistance in chronic myeloid leukemia; additionally, combined with MEK inhibitors AZD6244, PD98059 and trametinib in KRASMT pancreatic and colon cancer cells, it increases the suppression of tumor growth [39,40]. These results confirm the cross-talk between STAT3 signaling and RTKs. Some phenomena suggest that STAT3 is activated through cross talk between RTKs. Upon MEK inhibitor treatment, c-MET, but not IL-6, upregulates phosphorylation of STAT3. The combination of JAK1/2 and c-MET inhibitors increased the effect of MEK inhibition in KRASMT xenograft models [41]. In addition, EGFR and IL-6 signaling could in turn activate STAT3 after treatment with erlotinib [35]. Clearly, a combination of STAT3 pathway inhibitors and RTKs may offer a promising strategy to improve chemotherapeutic efficacy.

Drug-sensitive, oncogene-addicted cancer cells secrete factors that contribute to drug resistance via feedback mechanisms. For example, the secretion of IL-6 in turn stimulates the STAT3 pathway and mediates resistance to PI3K inhibitors [42]. IL-11, a member of the IL-6 family, is upregulated in cisplatin-stimulated cancer-associated fibroblasts, and then IL-11 activates the STAT3 signaling pathway, leading to lung adenocarcinoma cell resistance to cisplatin [43]. DeClerck and colleagues revealed the relationship between environment-mediated drug resistance (EMDR) and STAT3. In the presence of soluble IL-6 receptor (sIL-6R), IL-6 effectively activated STAT3 in neuroblastoma and prevented drug-induced apoptosis in neuroblastoma in a STAT3-dependent manner. Furthermore, monocytes, non-myeloid cells and Treg cells were part of the reciprocal loop of STAT3 activation [44]. IL-6 can be involved in regulating leukemic stem cell chemoresistance in acute myeloid leukemia (AML) via osteopontin (OPN) autocrine/paracrine signaling. AML cell proliferation and apoptotic inhibition were mediated through the AKT/VEGF/STAT3/CXCR4/IL-6 loop under three-drug (daunorubicin, cytarabine and idarubicin) treatment [45]. Another cytokine, IL-10, causes STAT3 phosphorylation to decrease the sensitivity of B16 melanoma cells to chemotherapeutic agents through an autocrine loop [46]. IL-10RA upregulation drives the resistance to crizotinib in anaplastic large cell lymphoma (ALCL) through an autocrine positive feedback loop. The IL-10/IL-10RA signaling pathway can reverse the effects of crizotinib-inhibition on STAT3 activity. Activated STAT3 further binds to the transcriptional start sites of IL-10/IL-10RA/IL-10RB in ALCL cells [47]. Trastuzumab treatment rendered resistance to itself, facilitated epithelial-to-mesenchymal transition and enhanced metastatic potential in gastric cancer cells through an IL6/STAT3/Jagged-1/Notch positive feedback loop [48]. MicroRNAs that regulate STAT3 activation are additional molecular mediators that contribute towards chemotherapeutic resistance and tumorigenicity. The IL-6R/STAT3/miR-34a feedback loop was required for maintenance of the mesenchymal phenotype and promoted invasion and metastasis in colorectal cancer [49]. Similarly, an IL6R/STAT3/miR-204 feedback loop was correlated with the cisplatin resistance of epithelial ovarian cancer cells [50]. These reports indicate that miRNA-dependent regulation of the STAT3 axis could provide a chance to overcoming the chemoresistance of cancer patients.

In addition, STAT3 on Tyr705 correlates with Burkitt lymphoma (BL) chemoresistance. Zhong’s group revealed that STAT3 confers chemoresistance to cells by mediating the antioxidant feedback [51]. In response to reactive oxygen species (ROS) after chemotherapy, STAT3 is phosphorylated and increases the expression of glutathione peroxidase (GPx1) and superoxide dismutase 2 (SOD2), thus reducing the level of ROS. The reduction of ROS is related to multidrug (CODOX, cyclophosphamide, vincristine and doxorubicin) resistance in BL cells [51].

## 3. STAT3 Activation by Radiation

Radiotherapy, alone or in combination with surgery and chemotherapy, is an indispensable link in comprehensive cancer treatment. Radiotherapy causes DNA damage directly or indirectly through generation of ROS and induces various forms of cancer cell death, including apoptosis, autography, mitotic catastrophe, necrosis and senescence. Radioresistance of tumor cells is one of the main biological factors that affect clinical outcomes [52,53,54,55]. Accumulating evidence indicates a link between STAT3 and radioresistance [56,57,58,59,60]. STAT3 Y705 activation occurs in a dose- and time-dependent manner upon radiation, mediates hepatobiliary malignancies and maintains stem cell self-renewal and tumorigenic potential [61,62,63,64,65]. STAT3 correlates with resistance to radiotherapy in HER2-positive breast cancer tissue, and radiation resistance in breast cancer stem cells is strongly associated with a high expression of STAT3 [66,67]. Potential STAT3 inhibitors can decrease the production of ROS and down-regulate phosphorylation of STAT3 and Bcl-2, restoring sensitivity to radiation, which offers an effective approach for treating triple-negative breast cancer cells [68]. Treg cells can regulate resistance to radiation in head and neck cancer. STAT3 antisense oligonucleotide (ASO) inhibited the phosphorylation of STAT3. ASO, in combination with radiation, decreased the population of M2 macrophages, Treg cells and myeloid-derived suppressor cells and resulted in potential antitumor immune responses [69]. Constitutive phosphorylation of STAT3 Tyr705 is present in B-1 lymphocytes and contributes to radioresistance. The microenvironment of the peritoneal cavity is important for B-1 cell radiation-induced apoptosis (RiA) with high levels of IL-6Rα and IL-10RI. These cytokines are a requirement for B-1 cell radioresistance via activation of STAT3 [56,70]. STAT3 Ser727 phosphorylation is also associated with radioresistance and has been reported to correlate with shortened overall survival and progression-free survival of GBM (glioblastoma) patients [71,72]. Table 1 lists the correlation between STAT3 and chemo(radio)sensitization through the modulation of the STAT3 pathway [59,67,68,73,74,75,76,77,78,79,80,81]. 

The development of radioresistance following radiation therapy coincides with the radiation-induced appearance of pSTAT3, epithelial–mesenchymal transition (EMT) and self-renewal. IL6/STAT3/TWIST signaling pathway activation mediates esophageal squamous carcinoma EMT, whereas inactivation of STAT3 by an IL-6 inhibitor or siRNA-mediated downregulation of Twist enhances cancer cell apoptosis and reverses radiation-induced EMT [80]. The STAT3/Slug axis is also connected to EMT phenotypes and cancer stemness that contribute to radioresistance in glioblastoma [82]. Upregulating ALDH1 expression can also induce EMT-associated CSC-like properties and lead to radioresistance via STAT3 activation [83]. Park and colleagues identified signaling as a mechanism for CRC (colorectal cancer) stem cell persistence and radioresistance [78]. Lip-FLLL32 sensitized pancreatic CSCs to chemotherapy and radiotherapy both in vitro and in vivo [7]. ROS have been demonstrated in IR-induced EMT. Some reports describe higher levels of phospho-STAT3 and lower levels of ROS than those in radiosensitive cancer cells [68,84]. Radiation also leads to DNA damage. Several studies report that STAT3 signaling pathway activation is involved in DNA repair. For example, high expression of MRE11, a core protein of the MRN (MRE11/RAD50/NBS1) repair complex, enhances breast cancer cell proliferation and invasion and promotes DNA repair through STAT3 [85]. DNA repair-associated molecules, such as ATM, ATR and BRCA1/2, are upregulated via IL-6/STAT3 signaling in radiation-resistant prostate cancer [86]. 

Together, these findings provide convincing evidence that STAT3 is activated by radiation and contributes to the cancer phenotype. Both STAT3 Y705 and S727 are potential targets for overcoming clinical radioresistance. Interrupting the STAT3 pathway by using STAT3 inhibitors, RNA interference and short hairpin RNA could sensitize radioresistant cancer cells and improve clinical outcomes.

**Table 1:** STAT3 contributes to chemo(radio)resistance in various carcinoma types.

Cancers	Cell Lines	Treatment	Inhibition of STAT3 Activation via Different Methods	In Vitro or In Vivo	Effects	Reference
Acute myeloid leukemia	KG-1/U937	DNR/Ara-C/IDR	Simvastatin/OPN siRNA	in vitro	Significantly decreases the viability of cells and the expression level of STAT3, which sensitizes cells to chemotherapy.	[45]
Anaplastic large cell lymphoma	SUP-M2	crizotinib	Stattic	in vitro	Inhibition of STAT3 can reverse the resistance to crizotinib in IR10RA-overexpressed cells.	[47]
B-1 lymphoma	B-1 cells	RT	STAT3^−/−^ mice	in vitro and in vivo	In STAT3^−/−^ mice, B cells are more susceptible to radiation-induced apoptosis.	[56]
Lung cancer	A549/H358/H157	RT	Niclosamide	in vitro and in vivo	Blocks IR-induced activation of the STAT3/Bcl-2/Bcl-xL pathway and enhances apoptosis.	[59]
	A549	RT	AZD0530	in vitro	Inhibits cell migration by blocking Src and enhances sensitivity to IR.	[73]
	A549	RT/cisplatin	shRNA	in vitro and in vivo	Enhances radiosensitivity both in vitro and in vivo and enhances chemosensitivity in vitro.	[74,81]
	HCC2429	RT	TG101209	in vitro and in vivo	Inhibits STAT3 activation and survivin expression, induces apoptosis and decreases proliferation.	[75]
	PC-9	Erlotinib	STAT3 siRNA/STAT3 shRNA	in vitro and in vivo	Inhibition of STAT3 feedback sensitized lung adenocarcinoma to MEK inhibition.	[35]
Breast cancer	MCF-7	RT	Xanthohumol	in vitro	Suppresses MDR1, EGFR and STAT3 expression, while increasing death receptor (DR)-4 and DR5 expression, and restores sensitivity to IR and doxorubicin.	[77]
	MDA-MB-231/MDA-MB-468	RT	Niclosamide/STAT3 shRNA	in vitro and in vivo	Niclosamide inhibits STAT3 and Bcl-2 and increases ROS generation in vitro and in vivo; it is identified as a radiosensitizer. shRNA of STAT3 sensitizes breast cells to IR.	[68]
	SKBR3	RT	Lapatinib/S3I-201	in vitro and in vivo	Inhibition of the HER2-STAT3-survivin axis increases sensitivity in SKBR3 cells.	[67]
	BT474	Trastuzumab	S3I-201	in vitro and in vivo	STAT3 inhibition significantly inhibits tumor growth and sensitizes breast cancer cells to trastuzumab.	[36]
Colorectal cancer	HCT116/LoVo	RT	JAK2 shRNA	in vitro and in vivo	Downregulation of JAK2/STAT3/CCND2 signaling to sensitize cells to radiotherapy and impair cancer stemness.	[78]
	HCT116/HCT29	5-FU/RT	Selumetinib	in vitro and in vivo	Increases mitotic catastrophe and apoptosis and decreases STAT3 activation and survivin expression; it also enhances sensitivity to radiotherapy in vivo.	[79]
	HCT116	Refametinib	4-IPP	in vitro	Inhibition of 4-IPP sensitizes cancer cells to refametinib.	[38]
Esophageal carcinoma	ECa109	RT	AG490	in vitro	Reverses the IR-induced EMT phenotypes and gene expression via regulation of the IL-6/STAT3/Twist signaling pathway. Attenuates radioresistance under AG490 treatment.	[80]
	ECa109/TE13/KYSE150	RT	Stattic	in vitro and in vivo	Stattic inhibits STAT3 activation, downregulates HIF-1α and VEGF expression; and confers radiosensitivity in vitro and in vivo.	[87]
	ECa109/TE13	RT	NSC74859	in vitro and in vivo	Sensitizes cells to radiotherapy in vitro and in vivo via inhibiting STAT3 activation and downregulation of HIF-1α and VEGF expression.	[88]
Glioblastoma	D456 GSCs	RT	Ibrutinib	in vitro	Combines with radiotherapy to disrupt tumor growth and mainly disrupts glioma stem cells by inhibiting bone marrow and X-linked (BMX)/STAT3 activation.	[64]
	GBM-R212	RT	STAT3 shRNA	in vitro	Reduces STAT3, decrease Slug expression and suppresses cell invasion; inhibits cancer stem cell properties and enhances radiotherapy.	[82]
	GSC-2/ GSC-11	RT	Stattic/WP1066	in vitro	Enhances radiosensitivity of GSC lines by inhibition of STAT3 activation, mainly impacting pSTAT3 on Serine727.	[72]
	U251/U87	RT	Cryptotanshinone/WP1066/S3I-201	in vitro and in vivo	Re-sensitizes radioresistant cells to radiotherapy by inhibition of STAT3; when combined with ERK1/2 inhibitors, it remarkably eliminates resistant cells and inhibits tumor regrowth.	[89]
Head and neck carcinoma	UMSCC-17B	RT	Stattic	in vitro and in vivo	Reduces STAT3-mediated HIF-1α expression in response to Stattic.	[90]
	UMSCC-22A/UMSCC-22B	RT	Linifanib	in vitro	Cell growth inhibition, G2/M cell cycle arrest and induction of cell death via apoptosis; it overcomes radioresistance by reducing pSTAT3 and expression of its target genes, e.g., cyclin D1, survivin.	[91]
	HNSCC-CD44(+)ALDH1(+) cell	RT	Cucurbitacin I	in vitro and in vivo	Combines with radiotherapy to suppress tumorigenesis and metastasis and also reduces CSC-like cells.	[76]
Hepatoblastoma	HepG2.2.15	RT	STAT3D		Suppresses RT-induced hepatitis B virus DNA replication and impairs hepatobiliary malignancies.	[62]
	PLC5/Huh-7/Sk-Hep1/Hep3B	RT	Sorafenib/STAT3 siRNA	In vitro and in vivo	Enhances radiation-induced apoptosis by inhibiting STAT3; it also downregulates Mcl-1, cyclin D1 and survivin expression, overcomes resistance to radiation in cells and suppresses tumor growth in vivo.	[92]

5-FU, 5-fluorouracil; Ara-C, cytarabine; Bcl-xL, B-cell lymphoma-extra large; DNR, daunorubicin; DR, death receptor; HIF-1α, hypoxia-inducible factor 1α; IDR, idarubicin; IR, irradiation; MDR1, multi-drug resistance 1; OPN, osteopontin; RT, radiation therapy; siRNA, small interfering RNA; shRNA, short hairpin RNA; STAT3D, dominant-negative mutant STAT3.

## 4. STAT3 Target Genes Impact Chemoresistance and Radioresistance

Activated STAT3 binds to DNA sequences (GAS element) in target genes and usually correlates with the enhanced expression of anti-apoptotic proteins, prevention of cell cycle arrest and promotion of cell proliferation [21]. A number of target genes, including Bcl-2 [68], Bcl-xL [5], Slug [82], Snail [93], survivin [94] and cyclin D1 [95], which have STAT3 binding sites in their promoters, regulate chemoresistance and radioresistance in a variety of cancer cells. Our group demonstrated that blocking the transcriptional activity of STAT3α by STAT3 isoform STAT3β sensitized esophageal squamous cell carcinoma (ESCC) cells to chemotherapeutic agents, namely, cisplatin and 5-FU both in vitro and in vivo [96]. The role of STAT3 target genes in chemo- and radioresistance is discussed in the following section.

### 4.1. Bcl-2 Family

Bcl-2, Bcl-xL and Mcl-1 are anti-apoptotic members of the Bcl-2 family and frequently dysregulated in various cancers. STAT3 has been shown to significantly enhance Bcl-2 and Bcl-xL promoter activity. Chromatin immunoprecipitation analysis indicates that activated STAT3 directly binds to the promoters of Bcl-2, Bcl-xL and Mcl-1 genes [97,98]. Hajime et al. further revealed the site of STAT3 that directly binds to the putative consensus sequences (position -92/-83) in the Mcl-1 promoter [99]. Thus, aberrant activation of STAT3 can activate Bcl-2 family proteins in various cancer cells [100]. Yu and colleagues suggested that STAT3 upregulated the expression of various target genes (Bcl-2, cyclin D1, c-myc) through a miR-197/CKS1B/STAT3 axis, which conferred chemoresistance to non-small-cell lung cancer [101]. Activated STAT3 increases RANTES (regulated upon activation, normal T cell expressed and secreted factor) and anti-apoptotic Bcl-2 and Bcl-xL expression levels that contributes to tamoxifen-resistance in breast cancer [97,100,102,103]. For cisplatin-induced mitochondrial-related apoptosis, knockdown of STAT3, by using siRNA, attenuated the expression of anti-apoptotic proteins Bcl-xL and Bcl-2 and increased the release of cytochrome C and the expression of Bax [102]. A similar result was found in ovarian cancer cells after treatment with the conditioned medium of cancer-associated fibroblasts [104]. In cisplatin-resistant ovarian cancer cells, NCX-4016 (nitro derivative of aspirin) treatment significantly decreases the protein levels of Bcl-2 and Bcl-xL and induces apoptosis in a time-dependent manner. The study further showed that the downregulation of STAT3 signaling could also inhibit tumor growth both in vitro and in vivo [103]. Doxorubicin-resistant chronic myeloid leukemia cells enhanced phosphorylation of STAT3 and dampened the tumor growth inhibition of TNF-α. STAT3 dephosphorylation can sensitize doxorubicin-resistant Dalton lymphoma to dendritic cell (DC)-derived TNF-α through the downregulation of Bcl-2 upon cucurbitacin I (STAT3 inhibitor) treatment in DC (chronic myeloid leukemia) patients [105]. (-)-Gossypol mainly disrupts Bcl-xL heterodimerization with Bax and Bad and overcomes the protection of prostate cancer cells by Bcl-xL. It also enhances the chemotherapy of docetaxel in inhibiting tumor growth and inducing apoptosis both in PC-3 cells and in xenograft model of prostate cancer [106]. 

The STAT3/Mcl-1 signaling axis is a target for overcoming resistance to BH3 mimetic ATB-737 (Bcl-2 inhibitor). Sorafenib, a multiple tyrosine kinase inhibitor, has been found to target Mcl-1 and synergize with ATB-737 to induce apoptosis in glioma cells [107]. However, (-)-gossypol also increases the expression levels of Mcl-1 [106], which may result in drug resistance. Sorafenib sensitizes prostate cancer cells to (-)-gossypol through the attenuation of Mcl-1 expression in vitro and in vivo. Using siRNA to downregulate Mcl-1, Lian et al.’s study also verified that prostate cancer cells were sensitized to (-)-gossypol [108]. 

Exposure of cells to IR (irradiation) can also increase Bcl-2 expression levels, which are associated with radioresistance [109]. In another study, radiotherapy resulted in the activation of STAT3 and Bcl-2 in triple-negative breast cancer. Persistent activation of STAT3 and Bcl-2 contributes to radioresistance [68]. Moreover, radioresistance was observed in DAB2 interactive protein-deficient prostate cells due to DNA double-strand break repair and the evasion of apoptosis with significantly higher levels of Bcl-2 and STAT3 [110]. Radiation induces the activation of STAT3 and increases the levels of Bcl-2/Bcl-xL in human lung cancer cells. Treatment with niclosamide can sensitize radioresistant lung cancer cells to ionizing radiation [59]. STAT3 and Mcl-1 are also induced by irradiation and are involved in the radioresistance of ESCC cells. A study further suggested that Mcl-1 inhibition was involved in the induction of apoptosis and the enhancement of DNA damage through SOCS1 overexpression [5]. Taking these results together, the upregulation of the Bcl-2 family proteins is the inescapable causative factor of chemo- and radioresistance.

### 4.2. EMT Regulators

Indeed, STAT3 is also important for initiating the epithelial-mesenchymal transition (EMT) program, which is associated with cancer therapy resistance [111]. Snail, Slug and Twist proteins are involved in regulating EMT [112]. Reports have shown that STAT3 can directly bind to the Slug promoters in a chromatin immunoprecipitation assay [82]. It also binds to Snail [93] and Twist1 [113] promoter and enhances their promoter activity. Reports have shown that STAT3/Slug was associated with GBM recurrence. STAT3 enhancement of tumor motility and EMT-like characteristics in GBM cells correlated with Slug expression. The efficacy of radiotherapy can be improved in vitro and in vivo by blocking STAT3/Slug signaling. Moreover, the blockage of STAT3/Slug also improves survival in GBM-R2I2 xenografts [82]. Slug was also upregulated in ovarian cancer and conferred chemoresistance to cells through the activation of STAT3 and the cross talk between p53/RAS signaling [114]. In other study, ovatodiolide (Ova), a diterpenoid isolate of *Anisomeles indica,* could overcome resistance to temozolomide (an alkylating agent) in glioblastoma. It reduced Slug, Vimentin, N-cadherin and β-catenin and also disrupted STAT3 signaling [115]. Moreover, Ova can significantly inhibit nasopharyngeal cancer cell tumor growth and enhance sensitivity to cisplatin in vivo. The study also found that Ova reduced Slug expression and inhibited EMT via abrogation of STAT3 signaling [116]. 

STAT3 upregulates the expression of Snail, contributes to temozolomide resistance in GBM and is associated with recurrent GBM tumors [117]. Another report also showed Snail/Slug-mediated chemoresistance to cisplatin in ovarian cancer cells [114]. Radioresistant head and neck squamous cell carcinoma cells showed high expression of Snail and Twist as the activation of STAT3 levels increased [76]. Increased expression of Snail was correlated with a poor prognosis in CRC patients. CRC cells that overexpressed Snail were also found to be more resistant to 5-FU [118]. Rectal cancer cells were resistant to ionizing radiation and 5-FU treatment due to the activation of STAT3 and the TGF-β/Smad signaling pathway. Treatment with metformin increased the sensitivity of rectal cancer cells by increasing apoptotic cell death as well as by downregulating Snail and Twist [119]. 

Twist basic helix loop helix transcription factor 1 (Twist1), a regulator of EMT, is upregulated in cisplatin-resistant ovarian cancer cells via STAT3 activation [120]. Inhibition of the IL6/STAT3/Twist signaling pathway could be a useful strategy to reverse radiation -induced EMT and radioresistance in ESCC [80]. Moreover, the inhibition of STAT3 activity and Twist1 transcription could suppress EMT and inhibit tumor progression and chemoresistance in ovarian cancer and renal cancer cells [113]. Wu et al. reported that DAB2 interactive protein suppressed the expression of Twist1 and the activation of STAT3. The report also demonstrated that Twist1 and STAT3 were crucial for the pirarubicin chemoresistance and tumor recurrence in non-muscle invasion bladder cancer, and this result could be reversed via DAB2 interactive protein [121].

### 4.3. Survivin

Survivin is an inhibitor of the apoptosis protein family, and its aberrant expression correlates with a poor prognosis and contributes to chemo(radio)resistance [122]. STAT3 is a potential transcriptional regulator of the survivin gene and binds to the survivin prompter at sites -264 to -256 [94]. Activation of STAT3 and survivin expression also confers resistance to chemotherapeutic agents (5-FU or cisplatin) in gastric cancer [123], hepatocellular carcinoma [124], NSCC [125] and ovarian cancer [104]. Survivin inhibitor MX106 effectively overcomes paclitaxel resistance in ovarian cancer cells [126]. In one study, STAT3 inhibition downregulated the expression of Bcl-xL, cyclin D1 and survivin, and induced apoptosis in a hepatocellular carcinoma xenograft model. The study also demonstrated that STAT3 inhibition enhanced chemosensitivity to cisplatin [127]. 

STAT3/survivin signaling regulates a poor response to radiotherapy in HER2-positive breast cancer [67], ESCC [5] and lung cancer [75]. Treatment with linifanib resulted in the induction of cell death via apoptosis and reduced activation of STAT3. It also decreased the expression of cyclin D1 and survivin and overcame radioresistance of head and neck squamous cell carcinoma [93]. Furthermore, using an inhibitor of JAK2, which is upstream of STAT3, affected survivin expression and sensitized lung cancer to radiation in vitro and in vivo [75]. Thus, inhibiting the expression of pSTAT3 and survivin can be efficient in improving the response to chemo- and radiotherapy.

### 4.4. Cyclin D1

Cyclin D1 peaks during mid-G1 when growth factor-deprived cells re-enter the cell cycle. Previous reports demonstrate that cyclin D1 confers chemo- and radioresistance to several tumor cells [128,129,130]. Activated STAT3 increases cyclin D1 mRNA expression, and binds to the positions -984, -568, -239 and -27 in human cyclin D1 promoters [95]. Several studies reported that inhibition of the STAT3 signaling pathway reduced the expression of cyclin D1 and enhanced the drug sensitivity of the cancer cells, including gastric carcinoma [131], cholangiocarcinoma [132], bladder cancer [133] and hepatocellular carcinoma [134]. An antisense cyclin D1 sequence in head and neck squamous cell carcinoma (HSNCC) cells inhibits cell growth, induces apoptosis and enhances sensitivity to chemotherapeutic agents both in vitro and in vivo [135,136]. Furthermore, Muneyuki et al. revealed that the levels of pSTAT3 played a causative role in the overexpression of cyclin D1 and provided a poor prognosis in HNSCC [137]. The researchers also demonstrated that a dominant-negative STAT3 strongly enhanced the cellular sensitivity of HNSCC cells to 5-FU and radiation [138]. 

Cyclin D1 can be stimulated by low-dose ionizing radiation (LDIR) in human keratinocytes. Inhibition of cyclin D1 using siRNA can inhibit the LDIR-associated anti-apoptotic response and eliminate LDIR-induced adaptive radioresistance [139]. Radioresistant ESCC cells possessed the EMT characteristic with cyclin D1 overexpression. Inhibition of cyclin D1 by using siRNA significantly decreased the cell proliferation rate and resulted in G0/G1 arrest, and it also enhanced sensitivity to radiation and reversed EMT [140]. Pestell’s group investigated the effect of cyclin D1 on radiosensitivity in prostate cancer cells. Cyclin D1 promoted the clonogenic survival of LNCaP cells by 6-fold upon radiation. Cyclin D1 knockdown using shRNA resulted in a radiation-dose dependent precipitous decline in clonogenic survival. The data suggested that cyclin D1 promoted radioresistance in LNCaP cells [141]. Cyclin D1 has also been implicated in nasopharyngeal carcinoma (NPC), resulting in cell survival by the cyclin D1-dependent DNA repair machinery. Moreover, controlling cyclin D1 by knocking down insulinoma-associated protein1 enhanced the sensitivity of NPC cells to radiation both in vitro and in vivo [142].

### 4.5. Immunosuppressive Molecules

Activation of STAT3 is important for tumor-induced immunosuppression, which regulates the expression of immunosuppressive molecules [143]. Yu’s study showed that blocking STAT3 signaling with STAT3β or antisense STAT3 oligonucleotide activated dendritic cells (DCs) by inducing the proinflammatory mediators IL-6, regulated upon activation normal T cell expressed and secreted factor (RANTES) and interferon-inducible protein-10 (IP-10) in tumor cells [144]. Further, they revealed that the expression of MHC class II, CD80 and CD86 was increased in tumor-infiltrating DCs of STAT3-null mice [145]. Moreover, T cells, DCs and natural killer (NK) cells are immune cells that are directly involved in anti-tumor responses regardless of the tumor’s sensitivity to the STAT3 inhibitor [145]. STAT3 influenced the maturation of DCs involving Paxillin-3-forkhead (PAX3-FKHR)-expressing cells. PXA3-FKHR interacted with STAT3 to reduce the expression of MHC and generate immunoinhibitory secreted factors such as IL-10 in tumor cells. Thus, the altered transcription induced by the PAX3-FKHR-STAT3 complex suppressed local inflammatory and immunological responses [146]. Another study demonstrated that tumor-derived factors prevented immature myeloid cells from differentiating into mature DCs via STAT3 activation. Except for the decrease in the expression of molecules (CD40, CD86) for T cell activation, the expression of MHC class II proteins also decreased [147].

IL-10 and TGF-β have been demonstrated to be involved in the suppression of the anti-tumor immune response of Treg cells [148]. Studies have reported that STAT3 bound to the promoters of the encoding genes of IL-10 [149] and TGF-β [150]. STAT3 is a key regulator of the Treg cells phenotype with the expression of IL-10, TGF-β and FOXP3 of nucleophosmin/anaplastic lymphoma kinase (NPM/ALK)-carrying T cell lymphoma (ALK+TCL) cells. IL-10 from ALK+TCL cells displays immunosuppressive activity, inhibits CD3/CD28 antibody-stimulated peripheral blood mononuclear cells and impairs the activation of T lymphocytes [151]. CD4^+^ CD25^+^ regulatory T cells are an important population of immunosuppressive cells. Treg cells from 12B1 tumor-bearing animals suppressed the function of DCs in a TGF-β- and IL-10-dependent manner and were associated with the activation of STAT3 [148]. Suppressor of cytokine signaling 3 (SOCS3)-deficient CD4^+^ T cells have a lower type 2 T helper cell (Th2) immune response and considerably increase the expression level of IL-10 and TGF-β for Th3-like differentiation via the enhanced recruitment of STAT3 to their promoters [150]. 

STAT3 has been identified on the promoter of vascular endothelial growth factor (VEGF) and was found to promote the growth and metastasis of human pancreatic cancer cells [152]. Chronic lymphocytic leukemia B cells secrete VEGF and activate the VEGF receptor (VEGFR). VEGF–VEGFR signaling is associated with abundant phosphorylated-STAT3 in the cell nucleus, which conferred apoptotic resistance to these cells [153]. Recently, Ong and colleagues found that a novel STAT3 mutation (p.D427H, E616G, p.E616K and p.E696K) increased phosphorylation of STAT3 at Tyr705 in STAT3 mutants. Further, p.E616K regulated programmed death-ligand 1(PD-L1) expression via the binding of STAT3 to the promoter of PD-L1 in NK/T-cell lymphoma (NKTL). The team suggested that inhibitors of STAT3 and PD-L1 might be a promising therapeutic strategy for NKTL [154]. Castration-resistant prostate cancer (CRPC) cells increased the expression of PD-L1 which participated in tumor immune evasion under hypoxic conditions. Silencing STAT3 signaling can reverse the level of PD-L1 and enhance the susceptibility of CRPC cells to NK cell immunity [155].

## 5. The Alternative Splicing Product STAT3β

### 5.1. Antitumorigenic Potential of STAT3β 

In various cells and tissues, the ratio of STAT3α to STAT3β ranges from 3:1 to 10:1 at the mRNA level, and from 1:3 to 10:1 at the protein level. Biethahn and colleagues found that STAT3β was expressed at a high level in CD34+ cells by Western blot analysis [156,157,158]. Because of the low expression levels of STAT3β, its role remained elusive for a long time. Woude and colleagues reported that STAT3β had no effects on branching morphogenesis or invasion of leiomyosarcoma cells. However, STAT3β significantly inhibited cell growth in soft agar in a dose-dependent manner [159], while cell-specific STAT3β expression in macrophages reduced the invasion and cell mobility of breast cancer cells and significantly suppressed breast tumor growth [160].

Given a truncated C-terminal transactivation domain, transcriptional activation by STAT3β probably involves cooperation with other transcription factors, such as c-Jun and STRA13 [161,162,163]. STAT3β has more prolonged Y705 phosphorylation and nuclear retention kinetics than STAT3α, impacts STAT3α-dependent transcription and regulates a number of genes [21,164]. Cooperating with c-Jun, STAT3β increases Fas expression and the sensitization of melanoma cells to Fas ligand-induced apoptosis [161,165]. Zammarchi and colleagues revealed that the STAT3β switch leads to tumor regression in vivo. Furthermore, they revealed that STAT3β has its own specific set of target genes (such as Cyclin C, IL-8 and PEX1) and increases breast cancer cell death [166]. Transient transfection of STAT3β strongly inhibits transcriptional activation of c-Src dependent Bcl-xL [167]. As a potential antitumorigenic molecule, overexpression of STAT3β inhibits the transcriptional activity of STAT3α, down-regulates the anti-apoptotic gene Bcl-xL and the cell cycle-related gene cyclin D1, and suppresses the proliferation of lung cancer cells [168]. In breast cancer cells, STAT3β mediates cells’ growth inhibition and leads to cell cycle arrest and apoptosis. Further, Niu and colleagues showed that STAT3β can induce the expression of TRAIL (tumor necrosis factor-related apoptosis-inducing ligand), which is a tumor-specific apoptotic effector [169]. Intriguingly, the expression of STAT3β can rescue the embryonic lethality of STAT3 deficient mice and induce the expression of specific STAT3 target genes [33].

STAT3β has gained attention as a potent tumor suppressor and a favorable prognostic factor in ESCC with or without chemoradiotherapy, and it strongly correlates with longer overall survival and recurrence-free survival [96]. Recently, it has been found that STAT3β correlates with a favorable prognosis and prolonged survival, and also has a tumor-suppressive effect in acute myeloid leukemia via the regulation of its target gene, SELL [170]. Taking these findings together, STAT3β is a potential protective prognostic marker.

### 5.2. STAT3β Switch Modulates Chemo(Radio)therapy Sensitivity 

As mentioned earlier, STAT3β is a truncated form of STAT3α due to alternative splicing and could be of value if one could control STAT3 alternative splicing. Nevo-Caspi and colleagues show that the level of ADAR editing enzymes increases upon deferoxamine treatment in lymphoblastoid cells, elevating the level of editing in Alu sequences residing in STAT3 introns [171]. They further demonstrate that ADAR1 affects STAT3 alternative splicing by RNA editing, which increases the expression of STAT3β [172].

Few studies have focused on switching STAT3α to STAT3β, which contributes to the inhibition of cancer cell growth (Figure 2). As mentioned above, PCBP1can regulate the proportion of STAT3α/STAT3β. PCBP1 inhibits oral squamous cell carcinoma cell growth and the expression of Bcl-xL and survivin, and it reverses the function of STAT3α [172,173]. Recently, phosphorodiamidate morpholino oligomers (morpholinos) were targeted to a splicing enhancer, and then STAT3α was specifically switched to STAT3β, which led to apoptosis and cell cycle arrest in breast cancer cells [166]. The splicing reprogramming offers an effective therapeutic approach for cancer. Similar results were obtained by using STAT3α-to-β expression-switching oligonucleotides to inhibit breast cancer cell growth and migration [174].

STAT3 has been reported in chemoresistance and radioresistance. In contrast, STAT3β plays an essential tumor-suppressive role in cancer [19,175]. Phosphorylation of STAT3 Ser727 is related to intrinsic radioresistance. STAT3β lacks the C-terminal 55 amino acids (containing S727), which indicates that STAT3β may enhance sensitivity to radiation. The roles of STAT3β in conferring radiation sensitivity to cells are still unclear to date [71,176]. Currently, STAT3β has been shown to be involved in regulating chemoresistance. STAT3β-transfected gastric and breast cancer cells inhibited the invasion of gastric cancer cells, promoted apoptosis and improved sensitivity to chemotherapy [177]. Interestingly, STAT3β expression is correlated with overall survival and recurrence-free survival in esophageal squamous cell carcinoma (ESCC) patients with or without chemoradiotherapy and sensitizes tumor xenografts to chemotherapy (cisplatin and 5-FU) mainly by blocking the transcriptional activity of STAT3α [96]. Taken together, these findings indicated that STAT3β is a potential molecule for sensitization to chemo(radio)therapy in cancer patients.

## 6. STAT3 as a Therapeutic Target

**Figure 3:**
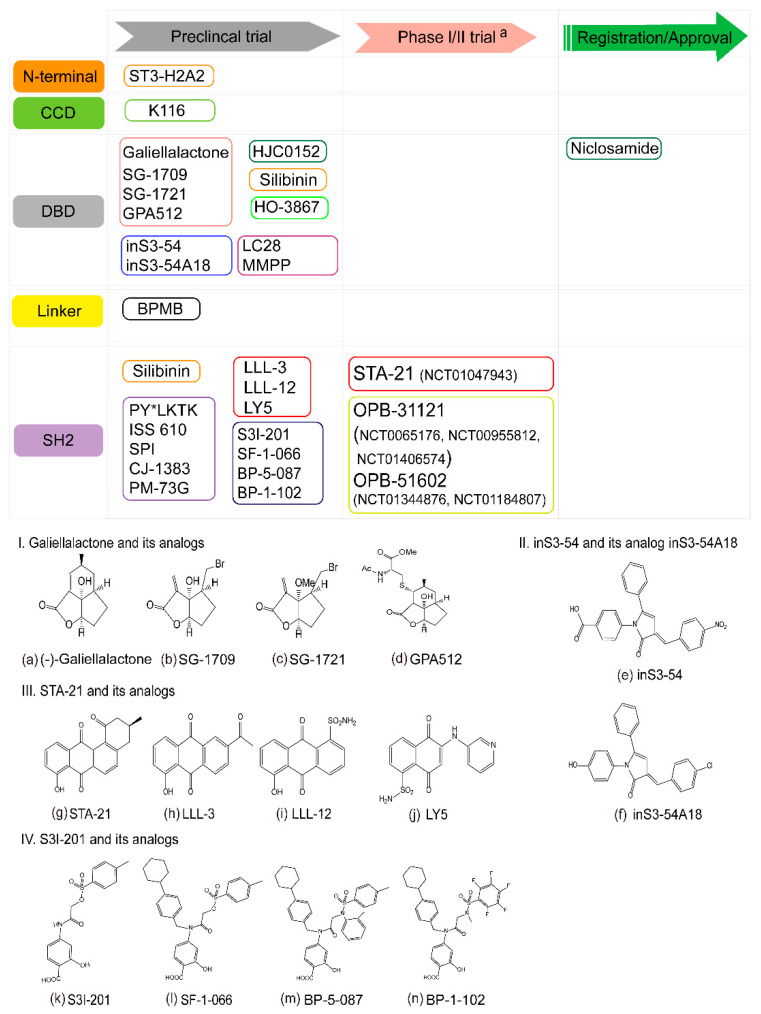
The current status of STAT3 inhibitors in (pre)clinical trials and their chemical structures. The N-terminal domain, DNA-binding domain (DBD), coiled-coil domain (CCD), linker domain and Src homology 2 domain (SH2) are components of the STAT3 structure. ^a^ Inhibitors of Phase I/II clinical trials registered at https://clinicaltrials.gov. (**a**–**d**), the structure of galiellalactone and its analogs. (**e**–**f**), the structure of inS3-45 and inS3-54A18. (**g**–**j**), the structure of STA-21 and its analogs. (**k**–**n**), the structure of S3I-201 and its analogs.

Early clinical studies demonstrated the promise of therapy that targets STAT3. However, no inhibitors that directly target STAT3 have been approved by the FDA for clinical application. In the following section, we focus on inhibitors that directly target STAT3 through its N-terminal domain, DNA-binding domain, linker domain and SH2 domain (Figure 3).

### 6.1. Targeting the STAT3 N-Terminal Domain

The N-terminal domain is essential for DNA binding, nuclear translation and protein–protein interactions, and a crystal structure is available for designing STAT3 NTD inhibitors [178]. A structural study has suggested that the overall fold of the domain is similar for different STATs. STAT4 structural data and sequence homology are used to design peptide analogs of STAT3 helix 2. The peptide (DTRYLEQLHQLY) was found to directly and specifically bind to STAT3, as determined by fluorescence resonance energy transfer (FRET) in cells expressing GFP-STAT3. The derivative potently inhibited survival and induced apoptosis in breast cancer cells, but not normal breast cells [179]. Furthermore, ST3-H2A2 (LDTRYLEQLHKLY) bound to both phosphorylated and unphosphorylated STAT3 in prostate cancer cells, decreased STAT3 DNA binding ability and induced apoptotic cell death [180].

### 6.2. Targeting the STAT3 DNA-Binding Domain

Blocking STAT3 by targeting the DBD domain could affect DNA binding ability and has the potential to inhibit STAT3-dependent gene transcription. Galiellalactone (Figure 3a), a natural product, was isolated from fermentations of the ascomycete strain A111-95. It can block IL-6 signaling by binding to the DNA binding sites of activated STAT3 dimers, but it does not affect the activation of STAT3 Tyr701 and Ser727 [181]. These anticancer agents have been investigated in prostate cancer cells and stem cell-like ALDH-positive prostate cancer cells both in vivo and in vitro. They led to a reduction in anti-apoptotic or cell cycle regulatory proteins through the inhibition of STAT3 signaling [182,183]. In a study on the effectiveness of galiellalactone in prostate cancer cells, Don-Doncow et al. showed that it acts as a cysteine reactive inhibitor and binds to one or more cysteines in STAT3 (Cys-367, Cys-468, Cys-542). The compound could lead to the inhibition of STAT3 binding to DNA [184]. Recently, it was shown to suppress the resistance of prostate cancer cells to enzalutamide [185], and its analogs SG-1709 (Figure 3b) and SG-1721 (Figure 3c) exhibited even greater effectiveness in blocking STAT3 signaling and inducing apoptosis. Interestingly, SG-1721 combined with radiotherapy showed enhanced apoptosis in triple-negative breast cancer cells [186]. To deal with the oral bioavailability of galiellalactone, the galiellalactone prodrug GPA512 (Figure 3d) was synthesized and shown to be able to reduce tumor growth in vivo through oral administration. The effect of GPA512 was similar to that of galiellalactone [187].

Niclosamide, an FDA-approved anthelmintic drug, as well as other small-molecule inhibitors, inhibits STAT3 activity. In combination with radiation, it can overcome the radioresistance of lung cancer xenografts [59]. Even though it potently inhibits the activation and transcriptional function of STAT3, its poor aqueous solubility and bioavailability limit its clinical development. A series of novel O-alkylamino-tethered niclosamide derivatives have been designed, with one, denoted HJC0152, being able to suppress breast cancer cell tumor growth in vivo. Its excellent aqueous solubility means that it can be taken orally in clinical cancer therapy [188].

Structure-based drug design and computational docking have been used for screening small-molecule inhibitors that target the DNA-binding domain of STAT3. The DBD-binding drug inS3-54 (Figure 3e) selectively inhibits the DNA-binding activity of STAT3, but it has off-target effects. The same team further designed an improved lead compound (inS3-54A18, Figure 3f) that not only binds to DBD directly, but also inhibits the expression of downstream STAT3 target genes. Its oral availability and anti-tumor effects in lung xenograft models argue against the previously thought “undruggable” nature of a DBD [189,190]. Ligand-based pharmacophore models of STAT3 inhibitors are being used in combination with a 3D shape- and electrostatic-based drug approach. LC28 is identified as a STAT3 DBD inhibitor through the pharmacophore of the inS3-45 analog A18. The compound inhibits the survival of cisplatin-resistant ovarian cancer cells [191]. An LC28 analog, MMPP exhibits the strongest binding affinity to STAT3 and binds selectively to the DBD, especially to T456. MMPP effectively inhibits STAT3 in vitro and in vivo and induces G1-phase cell cycle arrest and apoptosis [192]. On the basis of the conjugation of a diarylidenyl-piperidone (DAP) backbone to a nitroxide precursor, Kellie identified a novel small-molecule inhibitor, HO-3867, a curcumin analog that directly interacts with the STAT3 DNA-binding domain. HO-3867 has been studied in ovarian and pancreatic cancer cells, and in an ovarian cancer xenograft mouse model [193,194,195]. A molecular dynamics simulation was used for virtual ligand screening of a chemical library, and Buettner et al. found that C48 is a STAT3 inhibitor that selectively binds to Cys468 of STAT3 and blocks phosphorylation of STAT3 to inhibit tumor growth in vivo [196]. 

### 6.3. Targeting the STAT3 Linker Domain

Researchers have found a small-molecule inhibitor of the STAT3 linker domain. Tatsuya et al. identified the benzoquinone derivative BPMB as a STAT3 inhibitor. BPMB could selectively inhibit proliferation in constitutively-activated STAT3 breast cancer cells. From an analysis by matrix-assisted laser desorption/ionization-mass spectrometry, Cys550 was found to be an important residue for crosslinking between STAT3 and BPMB. Cys550 could be a drug target for the development of irreversible STAT3 inhibitors [197]. Furthermore, Berg and colleagues identified catechol bisphosphate as a STAT5b inhibitor that exhibited a higher degree of selectivity than STAT5a, which was determined by the linker domain (position 566). Given the homology of the STAT family, it is extremely likely that the linker domain governs selectivity in a similar manner in STAT3 [198].

### 6.4. Targeting the STAT3 SH2 Domain

Phosphorylation of tyrosine residues in the SH2 domain of STAT3 is essential for receptor recognition as well as dimerization. In a search to identify small molecule inhibitors of STAT3, an SH2 domain-binding phosphopeptide PY*LKTK (Y* represents phosphotyrosine) was identified and formed inactive STAT3·PY*LKTK complexes. It has significant in vivo activity and suppresses v-Src transformation by blocking STAT3 signaling [20]. Since the first peptide inhibitor of STAT3 was identified, a number of small-molecule inhibitors that inhibit STAT3 have been demonstrated. On the basis on the lead compound PY*L (PY*LKTK tripeptide), Turkson found a selective and more potent peptide mimetic, denoted ISS 610. It disrupts STAT3 activity in vitro, inhibits cell growth and induces apoptosis in Src-transformed fibroblasts [199]. Derived from the STAT3 SH2 domain, peptide mimetic SPI has been tested in pancreatic, breast, prostate and non-small lung cancer cells. SPI was shown to have good effectiveness, including viability loss and apoptosis, in all cell lines tested [200]. Other peptides and phosphopeptide mimetics have been reported and used in cancer cells, such as CJ-1383 and PM-73G. Both can selectively inhibit STAT3. CJ-1383 induced breast cancer cell apoptosis in a dose-dependent manner, while inhibition of tumor growth by the phosphopeptide mimetic PM-73G occurred without apoptosis or changes in the expression of cyclin D1 or survivin in xenograft models [201].

High-throughput screening of chemical libraries and computational docking studies have been used to discover inhibitors of STAT3. Stattic (6-nitro-benzo(b)thiophene-1,1-dioxide 1) was found to bind to the STAT3 SH2 domain. Another study demonstrated that Stattic enhanced radiosensitivity [202,203]. STA-21 (Figure 3g), a natural product analog of tetrangomycin, was discovered via structure-based virtual screening to bind to the SH2 domain and form hydrogen bonds with Arg595, Arg609 and Ile634 [204]. Phase I/II clinical trials (NCT01047943, 13 January 2010) for STA-21 have been performed for the treatment of psoriasis [205,206]. Analogs of STA-21 were reported later, such as LLL-3 (Figure 3h) and LLL-12 (Figure 3i), which can suppress cancer cell proliferation and induce apoptosis [207,208,209]. Using in silico site-directed fragment-based drug design (FBDD), the small-molecule STAT3 inhibitor LY5 (Figure 3j), an analog of STA-21, was tested in colon cancer cells. LY5 inhibited STAT3 phosphorylation and suppressed colon tumor growth in vivo [210]. The results suggest that LY5 can be an effective agent in cancer patients with constitutive activation of STAT3, and represents a promising approach for overcoming drug resistance induced by feedback activation of STAT3.

S3I-201 (NSC 74859, Figure 3k) is a selective chemical probe inhibitor of STAT3 and binds to the STAT3 phosphotyrosine peptide (PY*LKT) with the potential to become a radiosensitizer for esophageal cancer radiotherapy [88,211]. S3I-201 alkylates multiple Cys residues of STAT3 (Cys 108, 259, 367, 542 and 687), and is identified as a non-selective alkylating agent due to its leaving group *O*-tosyl [212]. Molecular modeling, using S3I-201 as the lead compound, led to S3I-1757, which could interact with Y705 in the binding site of the SH2 domain and decrease nuclear phosphotyrosine STAT3 levels [213]. To optimize S3I-201, Steven et al. synthesized a large set of S3I-201 analogs, among which SF-1-066 (known as S3I-201.1066, Figure 3l) and SF-1-121 showed impressive potency in whole-cell viability assays, and SF-1-066 also induced an antitumor response in breast tumor xenografts [214,215]. Further improvement of the parent compound SF-1-066 led to BP-5-087 (Figure 3m), a highly potent and selective STAT3 SH2 domain inhibitor, and when used in combination with imatinib, it could reverse TKI-resistant CML [39]. BP-1-102 (Figure 3n), identified as an analog of SF-1-066 via computer-aided design, also exhibited similar effects in breast cancer xenografts [216]. BP-1-102 is orally bioavailable and detectable at the micrograms levels in tumor tissues, which is enough to inhibit tumor growth. Even though micromolar concentrations of BP-1-102 in plasma were detected, the blood levels exceeded the IC50 (inhibitory concentration 50%, 6.8 ± 0.8 μM) [216]. To reduce the pharmacokinetic lability of BP-1-102, SH-4-54 was designed as a STAT3 SH2 domain inhibitor, which showed better therapeutic efficacy than BP-1-102, and exhibited blood–brain barrier permeability and controlled glioma tumor growth [217].

More recently, OPB-31121 (Otsuka pharmaceuticals Co. ltd.) and OPB-51602, potent small molecule inhibitors of STAT3, were found to strongly inhibit STAT3 phosphorylation and lead to effective antitumor responses [218,219,220,221]. They interact with the SH2 domain of STAT3 with high affinity and inhibit STAT3 Y705 and S727 phosphorylation. Computational docking and molecular dynamics simulations were used to examine the binding of OPB-31121 to STAT3, and OPB-31121 bound to S636 and V637 residues without overlapping with other STAT3 inhibitors [221,222]. OPB-51602 can directly interfere with mitochondrial STAT3 and induce the formation of STAT3 proteotoxic aggregates that are lethal to cancer cells [223,224]. These two inhibitors show the most potential among STAT3 SH2 domain inhibitors to date, based on preclinical studies that describe antitumor ability against various cancers. Phase I/II clinical trials for both OPB-31121 and OPB-51602 have been completed. These include a Phase I clinical trial for OPB-31121 in advanced cancer and solid tumor in Korea (NCT00657176, 18 April 2008) [225] and the USA (NCT00955812, 12 February 2013) [226], a Phase I/II trial in patients with progressive hepatocellular carcinoma (NCT01406574, 8 June 2015), and a Phase I OPB-51602 clinical trial for hematological malignancies in Japan (NCT01344876, 8 June 2015) [221] and for refractory solid tumors (NCT01184807, 26 March 2014) [220].

### 6.5. The Challenge of STAT3 Inhibitors

Aberrant STAT3 activation is related to cell malignant transformation, tumor proliferation, differentiation and anti-apoptosis [175]. Thus, STAT3 is considered a promising target in the development of anti-tumor drugs. Clearly, an accumulating number of STAT3 inhibitors have been discovered, as mentioned above. Most of them are mainly in the preclinical stage for screening drug activity, with few pharmacological and toxicological studies in animals and few compounds used for clinical trial.

The peptide (DTRYLEQLHQLY) and ST3-H2A2 (LDTRYLEQLHKLY) provide an additional strategy in the discovery of anti-STAT3 therapeutic agents as a novel anticancer therapy. However, the mechanisms of the peptides’ interaction with the N-terminal domain of STAT3 still need to be investigated. The ”undruggable” nature of a DBD has been proven druggable in the case of STAT3. Inhibitors targeting the DBD have been in a minor development stage and will face challenges. In recent years, silibinin has been shown to have unique characteristics whereby it binds directly with high affinity to both the SH2 domain and DBD of STAT3, making it a promising lead candidate for designing novel and effective STAT3 inhibitors [227]. Although few small-molecule inhibitors of the STAT3 linker domain have been discovered, STAT3 mutants with a mutation in the linker domain exhibit very weak transcriptional activity [228]. The linker domain between the DNA-binding domain and the SH2 domain offers a new strategy for developing small-molecule inhibitors of STAT3. 

At present, the main focus is on STAT3 inhibitors that bind to the tyrosine phosphorylation site of the SH2 domain of STAT3. The general structure of the SH2 domain and the mode of phosphopeptide recognition are conserved. The site is rich in positively charged residues, which requires a high negative charge of small molecules [229]. Therefore, inhibitors for targeting the SH2 domain generally have large side effect and poor pharmacokinetic characteristics. Faced with this challenge, Zhang and co-workers recently identified allosteric sites and a modulator of the coiled-coil domain of STAT3 via AlloFinder. The team confirmed that K116 both inhibited STAT3 Tyr705 and promoted apoptosis in a dose-dependent manner [230]. 

In summary, small-molecule STAT3 inhibitors have antitumor activities and inhibit STAT3 phosphorylation but are yet to be approved for clinical cancer therapy. Inhibitors that target the SH2 domain of STAT3, such as STA-21, OPB-31121 and OPB-51602, have completed Phase I/II studies. These compounds are expected to progress in further clinical trials in the future and pave a new avenue for cancer therapy. The inhibitors targeting STAT3 may induce unexpected side-effects because of the repression of STAT3β, and regulators and drugs for the regulation of STAT3 alternative splicing remain to be investigated.

## 7. Conclusions and future perspectives

Chemoradiotherapy represents an important treatment for cancers. However, the intrinsic and acquired resistances of drug or radiation treatment limit its effectiveness in the clinic. Current evidence indicates that STAT3 is an oncogene, is constitutively activated in a variety of cancers, and regulates resistance to chemotherapy and radiotherapy. Signal transduction pathways influencing radiosensitivity mainly upregulate anti-apoptotic and cell cycle genes, induce EMT or modulate DNA repair [54]. Targeting the STAT3 pathway alone or in combination with other drugs can reverse resistance to chemotherapy or radiotherapy to significantly improve the effectiveness of therapy [4,35,36,48]. These findings show STAT3 to be a promising molecular target for the treatment of cancer. Inhibitors of different STAT3 domains have been identified via the screening of chemical libraries and computational docking. At present, few inhibitors have completed Phase I/II clinical trials. It is extremely urgent and important that we discover clinically useful inhibitors that target STAT3. 

Conversely, constitutively active STAT3 inhibits cell proliferation and invasion in PTEN-deficient cancer cells [231,232]. The anti-tumor effects of STAT3 have also been reported for p19ARF-negative hepatocytes [233]. As mentioned above, STAT3β functions as a tumor suppressor and sensitizes cancer cells and ESCC patients to chemotherapy. The conflicting functions of STAT3 in cancers further indicate that STAT3 inhibitors must be used cautiously and could lead to enhanced tumor progression. To date, the binding sites of STAT3 inhibitors have docked to the crystal structure of STAT3β. These inhibitors probably bind to both STAT3α and STAT3β, and the selective binding affinities have not been characterized. Because of the unique structures of STAT3α and STAT3β, drugs targeting the C-terminal domain can be developed. We hope that promising drugs that selectively target STAT3α are forthcoming and that these drugs can reduce the possibility of side effects.

Interestingly, the ratio of STAT3α/STAT3β impacts the fate of a tumor. Splicing mediators, oligonucleotides and morpholinos can switch STAT3α to STAT3β in several cancer cells. Hopefully, in the next few years, several drugs that induce the expression of STAT3β by affecting alternative splicing regulators and increase chemo- and radiotherapeutic efficiency will be successfully applied in the treatment of cancer patients.

## Abbreviations

AKT	protein kinase B	JAK	Janus kinase
Arg	arginine	KRAS	Kirsten rat sarcoma 2 viral oncogene homolog
ATM	ataxia telangiectasia mutated	lncRNA-SRLR	sorafenib resistance-associated lncRNA in RCC
Bax	Bcl2 associated X	MEK	mitogen-activated protein kinase kinase 7
Bcl-2	B-cell lymphoma 2	NKTL	NK/T-cell lymphoma
Bcl-xL	B-cell lymphoma-extra large	MLS	myxoid liposarcoma
BLACAT1	bladder cancer associated transcript 1	NTD	amino-terminal domain
BL	Burkitt lymphoma	NSCLC	non-small-cell lung cancer
CCD	coiled-coil domain	PCBP1	poly-C binding protein-1
CKS1B	cyclin-dependent kinase regulatory subunit 1B	PD-L1	programmed death ligand 1
CML	chronic myeloid leukemia	PI3K	phosphonosinol-3 kinase
CRC	colorectal cancer	PTEN	phosphatase and tensin homolog
CRT	chemoradiotherapy	RCC	renal cell carcinoma
CSC	cancer stem cell	ROS	reactive oxygen species
DAP	diarylidenyl-piperidone	RT	radiation therapy
DBD	DNA-binding domain	RTKs	receptor tyrosine kinases
DC	dendritic cell	Ser727	serine 727
EGF	epidermal growth factor	SELL	selectin L
EGFR	epidermal growth factor receptor	SH2	Src homology 2
EMT	epithelial-mesenchymal transition	STAT	signal transducer and activator of transcription
ESCC	esophageal squamous-cell carcinoma	TAD	transcriptional activation domain
FBDD	fragment-based drug design	TGF-β	transforming growth factor β
FN	Fibronectin	TKIs	tyrosine kinase inhibitors
FRET	fluorescence resonance energy transfer	Twist1	twist basic helix loop helix transcription factor 1
GAS	interferon γ-activated sequence	Val	valine
GBM	Glioblastoma	VEGFR	vascular endothelial growth factor receptor
HER2	human epidermal growth factor receptor 2	Tyr705	tyrosine 705
IL-6	interleukin-6	Ile	isoleucine
